# Supplementary material for: High-temperature probe of electron compressibility via asymmetric Coulomb drag
Source: Nat Commun. 2026 Feb 5;17:2393. doi: 10.1038/s41467-026-69086-9 (PMC12982821; doi:10.1038/s41467-026-69086-9)
Supplement: Supplementary file 1 — Supplementary Information [file 41467_2026_69086_MOESM1_ESM.pdf]

Supplementary Information for

High-temperature probe of electron compressibility via asymmetric Coulomb drag

Outlines

1. Schematic of the fabrication process of the drag devices.

2. Optical images of the devices tested.

3. Optical images of the fabrication process of the drag devices.

4. Different dual-gated Coulomb drag device configurations and their corresponding drag signals.

5. Transfer curves of the graphene and MoS_2 layer at different temperatures.

6. Ohmic contact of the MoS_2 layer at different temperatures.

7. Ohmic contact of the graphene layer at different temperatures.

8. Contact resistance of the graphene layer as a function of temperature and gate voltages.

9. Contact resistance of the MoS_2 layer as a function of temperature and gate voltages.

10. Band edge of the window-contacted MoS_2 layer.

11. Impact of bridge balance on the accuracy of drag signal measurements.

12. Identification of the effective Coulomb drag region.

13. The location where the maximum value of R_{drag} occurs.

14. The leakage current I_g between the drive and drag layers.

15. Linear response of drag voltage to drive current at low temperature.

16. Onsager reciprocity in the drag system.

17. Determination of carrier density by classic Hall effect.

18. Hall carrier density and Hall mobility of each layer for different temperatures and gate voltages.

19. Field-effect mobility of each layer for different temperatures and gate voltages.

20. Onsager reciprocity relation of magneto-drag resistance.

21. Magneto-drag behavior in semiconductor-semimetal bilayers at 5 T and 30 K.

22. Drag oscillations versus $1/B$ show the same spacing as graphene's SdH oscillations.

23. The visible oscillations of the magneto-drag signal at high temperatures in sample S24.

24. The drag response for samples with different spacer thickness d.

1. Schematic of the fabrication process of the drag devices.

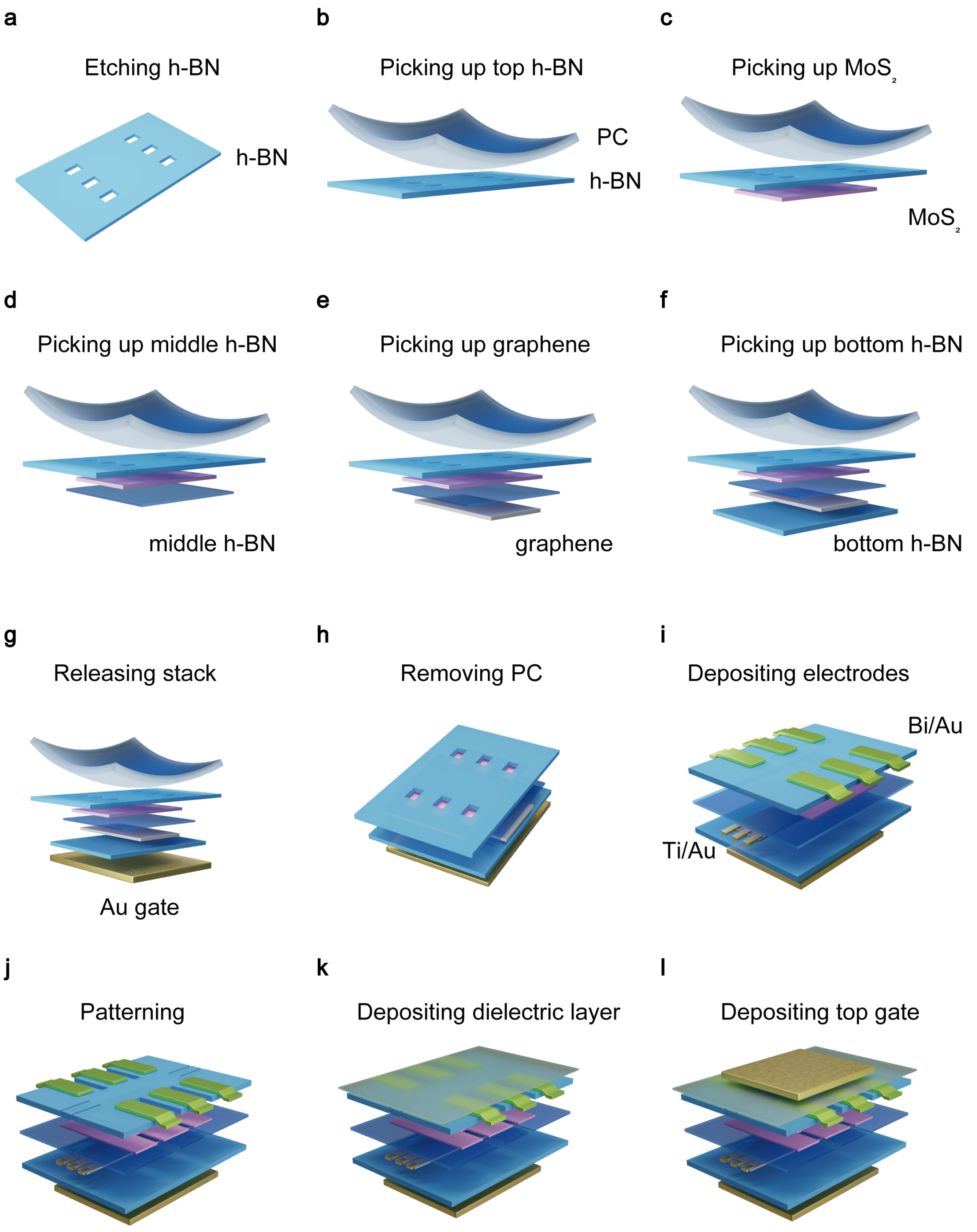

Supplementary Figure 1. Schematic of the fabrication process of the drag devices. (a)-(f) Using windowed h-BN as the top layer and polymer carbonate (PC) as the adhesive layer, sequentially pick up MoS_2, a thin layer of h-BN, and graphene, and then transfer to the bottom h-BN for sample encapsulation. (g) The h-BN/MoS_2/h-BN/graphene/h-BN stacks are placed onto Au or graphite bottom gates, followed by the releasing of the PC stamp. (h) Residues of PC are removed by solvent of chloroform. (i) Ti/Au electrodes as well as Bi/Au electrodes are deposited by e-beam evaporator

and thermal evaporator, respectively. (j) The heterostructures are then patterned into Hall bars via plasma etching. (k) Deposit a dielectric layer on the surface of the stack by ALD. (l) Top gate is fabricated over the channel region.

2. Optical images of the devices tested.

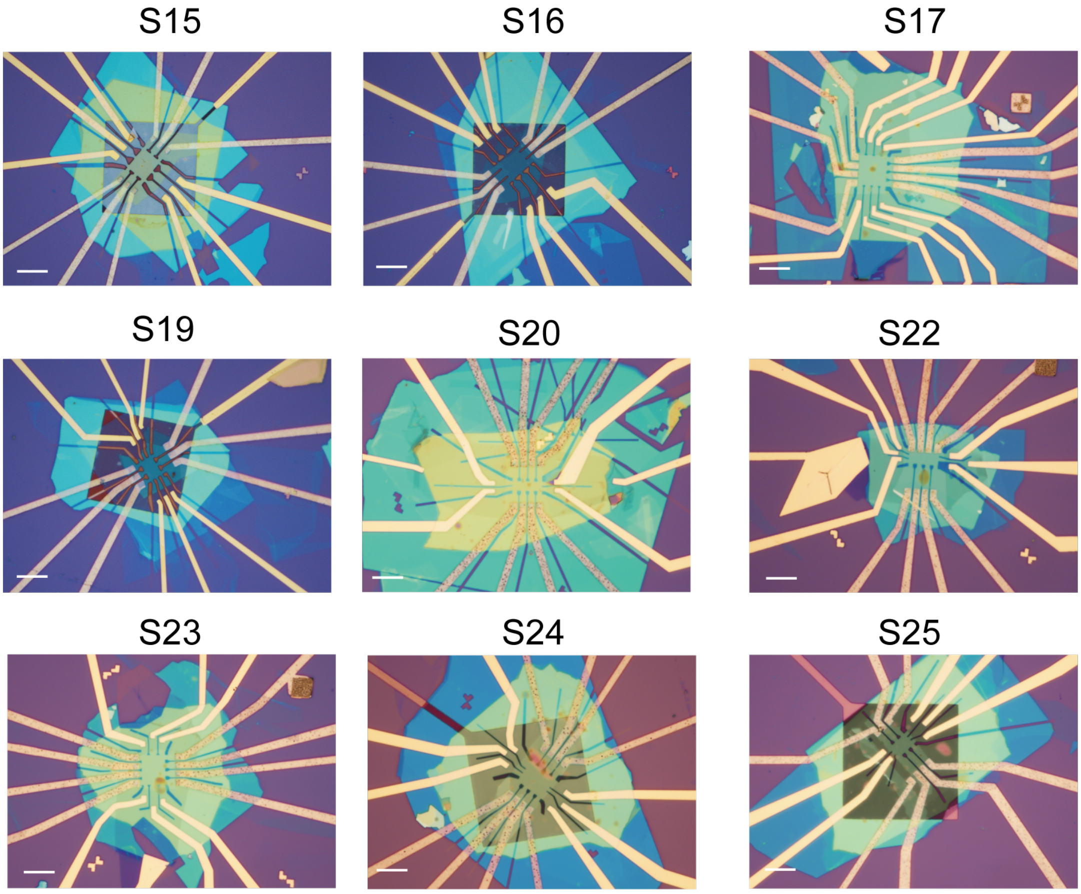

Supplementary Figure 2. Optical images of the devices tested. Roster of the optical images of bilayer MoS_2 –monolayer graphene drag devices. The white scale bars in the bottom left corners represent 10 μm.

3. Optical images of the fabrication process of the drag devices.

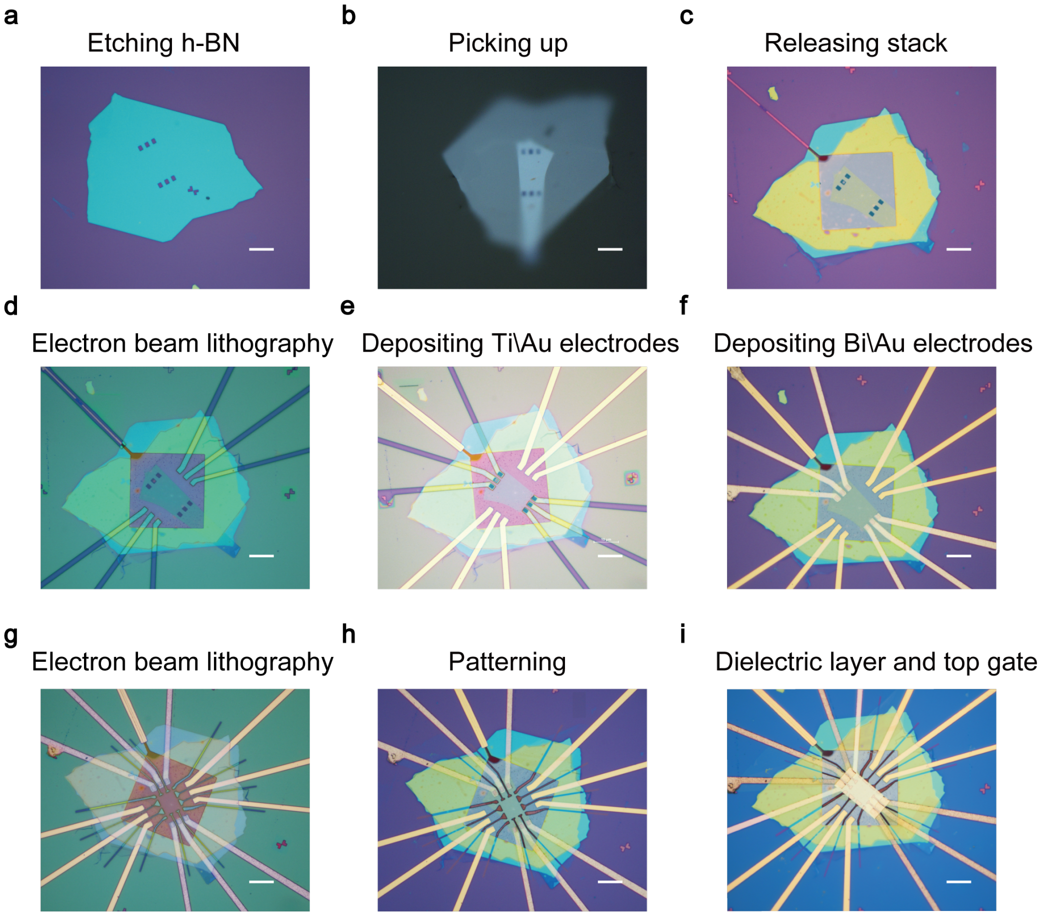

Supplementary Figure 3. Optical images of the fabrication process of the drag devices. (a)-(b) Using windowed h-BN as the top layer and polymer carbonate (PC) as the adhesive layer, sequentially pick up MoS_2, a thin layer of h-BN, and graphene, and then transfer the bottom h-BN for sample encapsulation. (c) The h-BN/MoS_2/h-BN/graphene/h-BN stacks are placed onto Au or graphite bottom gates, followed by the releasing of the PC stamp. Then, PC are removed by solvent of chloroform. (d)-(f) Ti/Au electrodes as well as Bi/Au electrodes are deposited by e-beam evaporator and thermal evaporator, respectively. (g)-(h) The heterostructures are patterned into Hall bars using electron beam lithography and plasma etching. (i) Deposit a dielectric layer on the surface of the stack by ALD. And top gate is fabricated over the channel region. The white scale bars in the bottom right corners represent 10 μm.

4. Different dual-gated Coulomb drag device configurations and their corresponding drag signals.

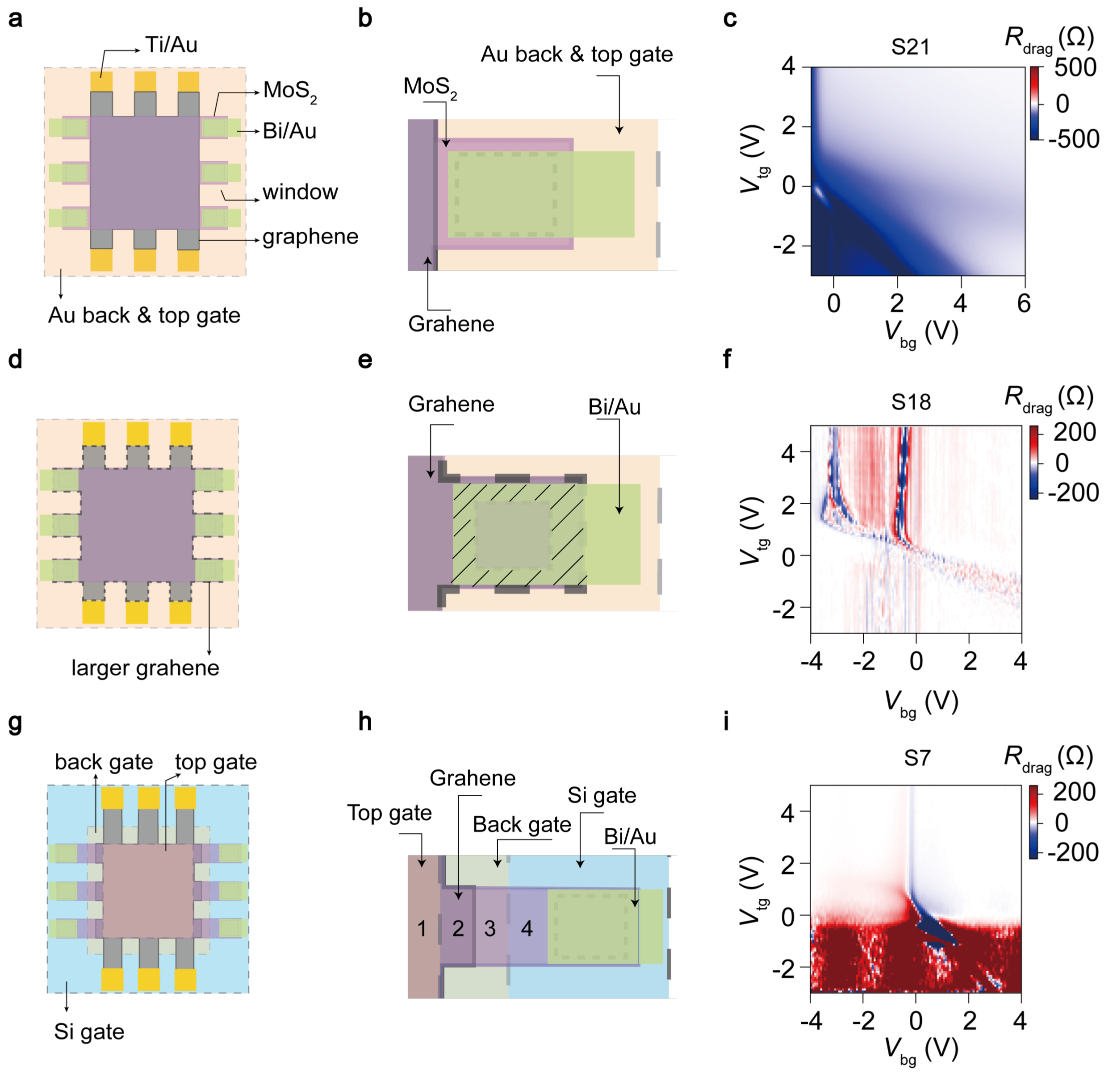

Supplementary Figure 4. Three kinds of fabricated drag devices and their associated drag signals. (a) The schematic of a typical MoS_2-graphene drag device (used in the manuscript). This device configuration consists of a large local Au (or graphite) back gate and top gate. Bi/Au electrodes are used for Ohmic contact for MoS_2 and Ti/Au electrodes are used for one dimensional Ohmic contact for graphene. (b) and (c) show the corresponding zoomed-in schematic top view of the Bi/Au electrode and measured drag signals as a function of V_{bg} and V_{tg}, respectively. (d) The schematic illustrates a drag device with the graphene layer larger in size than the MoS_2 layer. The dashed line represents graphene. (e) and (f) are the corresponding zoomed-in schematic top view of the Bi/Au electrode and measured drag signals as a function of V_{bg} and V_{tg}, respectively. (g) The schematic depicts a drag device utilizing a silicon gate for tuning the carrier density in the contact region of MoS_2, while the overlapped area represents the top and bottom gates. (h) and (i) show the corresponding zoomed-in schematic top view of the Bi/Au electrode and measured drag signals as a function of V_{bg} and V_{tg},

respectively. Overall, the second configuration (d) will prevent tuning the contact resistance of MoS_2 efficiently due to the screening induced by graphene and result in chaotic drag signals as shown in (f). For the third configuration (g), the contact of MoS_2 is divided into four distinct regions due to the possible misalignment of top and bottom gates, which will lead to spurious drag signals presented in (i). Based on the evident comparison of the measured drag signals, we select the first device configuration for further measurements and analysis.

5. Transfer curves of the graphene and MoS_2 layer at different temperatures.

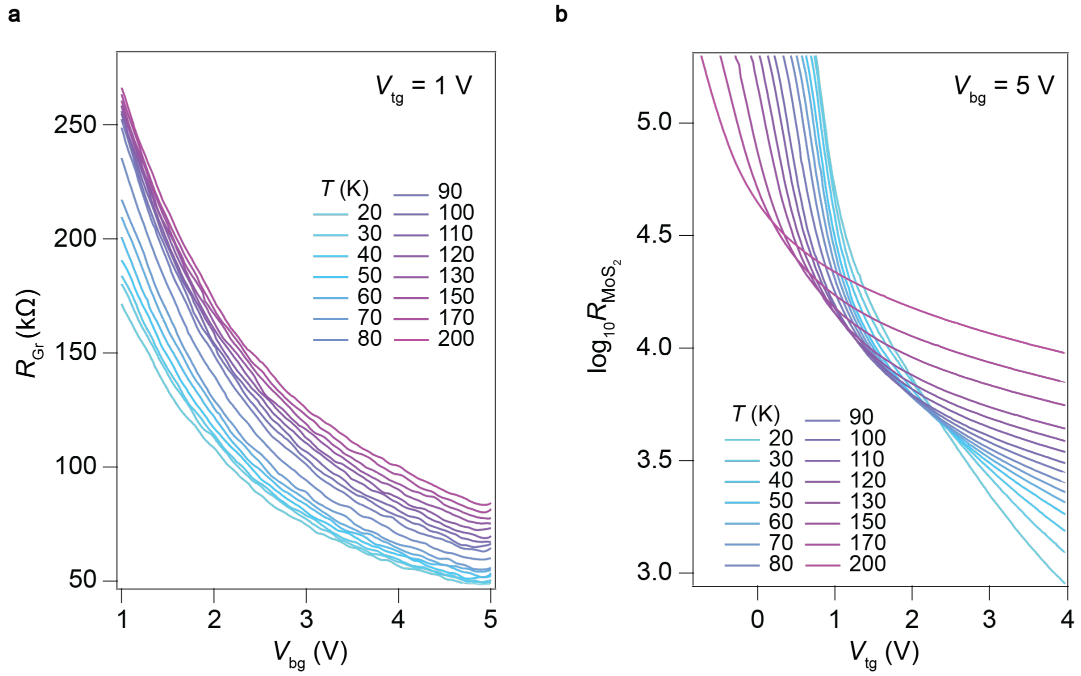

Supplementary Figure 5. The drain current of (a) monolayer graphene and (b) MoS_2 as a function of gate voltages for different temperatures, respectively.

6. Ohmic contact of the MoS_2 layer at different temperatures.

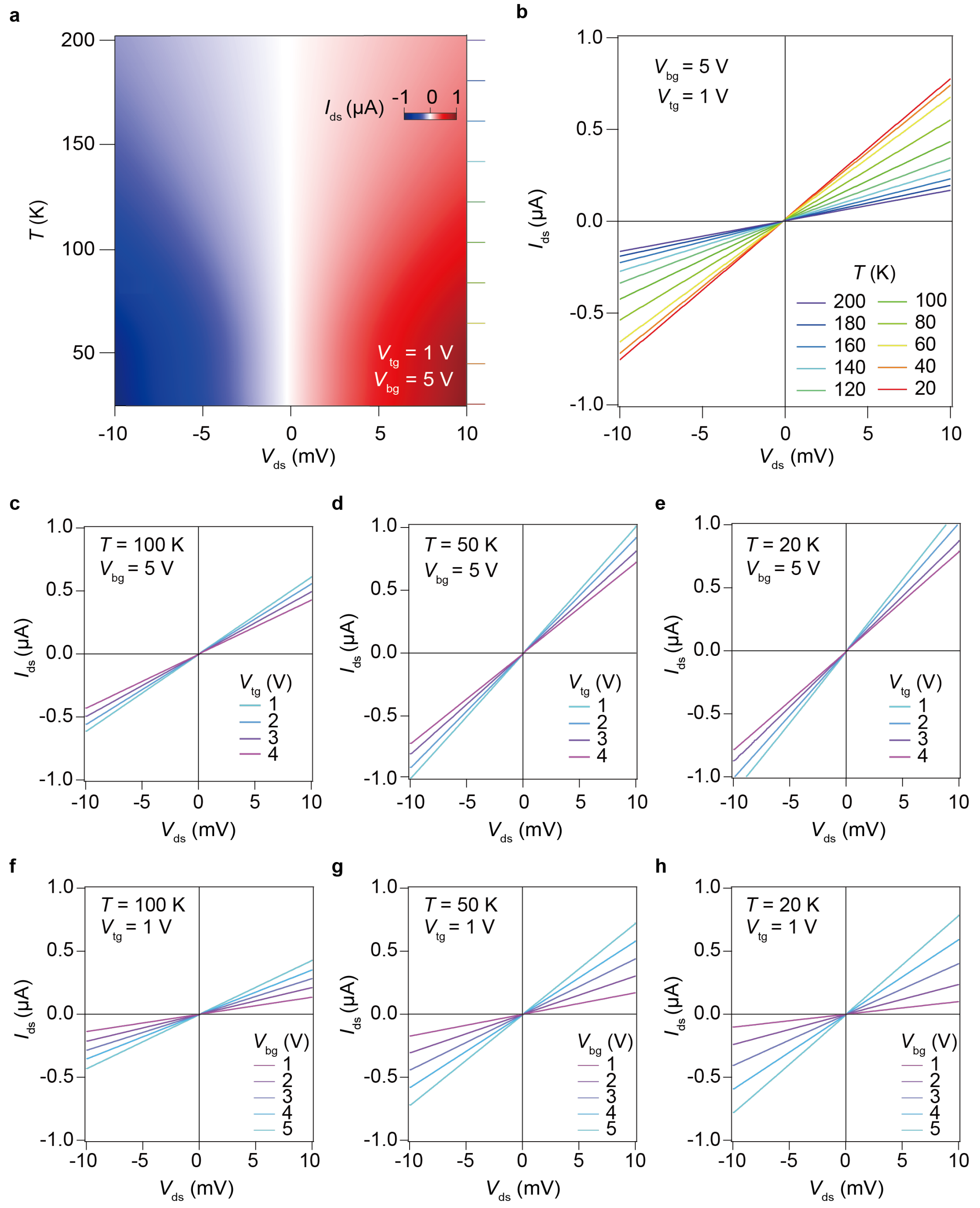

Supplementary Figure 6. Output curves of MoS_2 at different temperatures. The color map of the drain current in MoS_2 as a function of V_{ds} and temperature at V_{tg} = 1 V and V_{bg} = 5 V. (b) The *I*-*V* curves of MoS_2 at different temperatures extracted from (a). The *I*-*V* curves of MoS_2 with different V_{tg} at (c) T = 100 K, (d) T = 50 K and (e) T = 20 K, respectively. The applied bottom voltage is 5 V. The *I*-*V* curves of MoS_2 with different V_{bg} at (f) T = 100 K, (g) T = 50 K and (e) T = 20 K, respectively. The applied top voltage is 1 V.

7. Ohmic contact of the graphene layer at different temperatures.

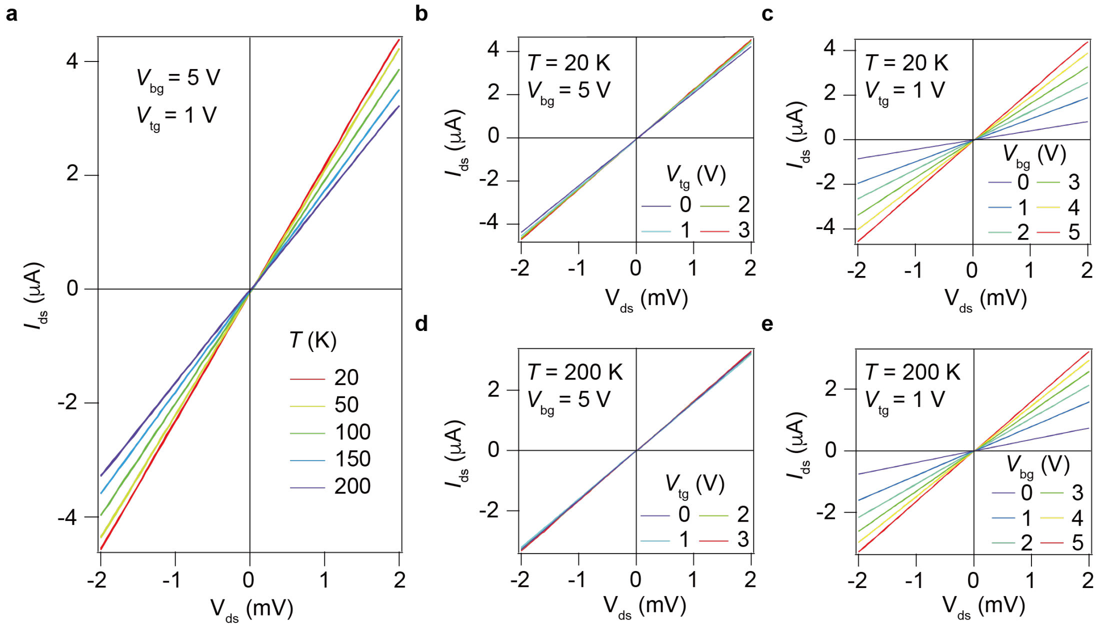

Supplementary Figure 7. Output curves of monolayer graphene at different temperatures. (a) The *I*-*V* curves of monolayer graphene at different temperatures for V_{tg} = 1 V and V_{bg} = 5 V. The *I*-*V* curves of monolayer graphene at *T* = 20 K for (b) V_{bg} = 5 V and (c) V_{tg} = 1 V, respectively. The *I*-*V* curves of monolayer graphene at *T* = 200 K for (d) V_{bg} = 5 V and (e) V_{tg} = 1 V, respectively.

8. Contact resistance of the graphene layer as a function of temperature and gate voltages.

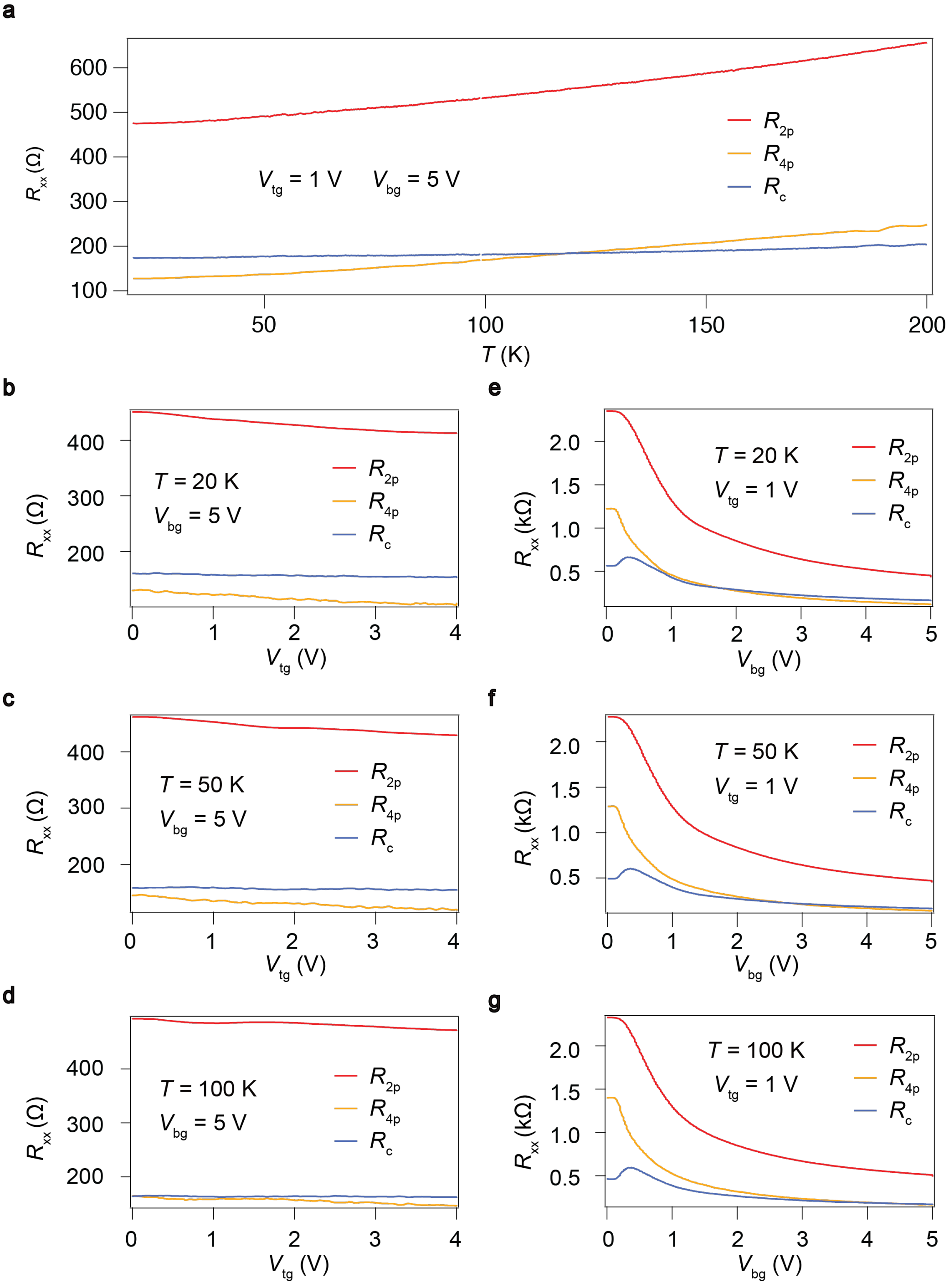

Supplementary Figure 8. Contact resistance of monolayer graphene at different temperatures and gate voltages. (a) Two-terminal resistance R_{2p}, four-terminal resistance R_{4p} and the extracted contact resistance R_c for graphene layer in sample-S21 as a function of temperature. The contact resistance R_c for graphene layer in sample-S21 as a function of V_{tg} at (b) $T = 20$ K, (c) $T = 50$ K, and (d) $T = 100$ K, respectively. The contact resistance R_c for graphene layer in sample-S21 as a function of V_{bg} at (e) $T = 20$ K, (f) $T = 50$ K, and (g) $T = 100$ K, respectively.

9. Contact resistance of the MoS_2 layer as a function of temperature and gate voltages.

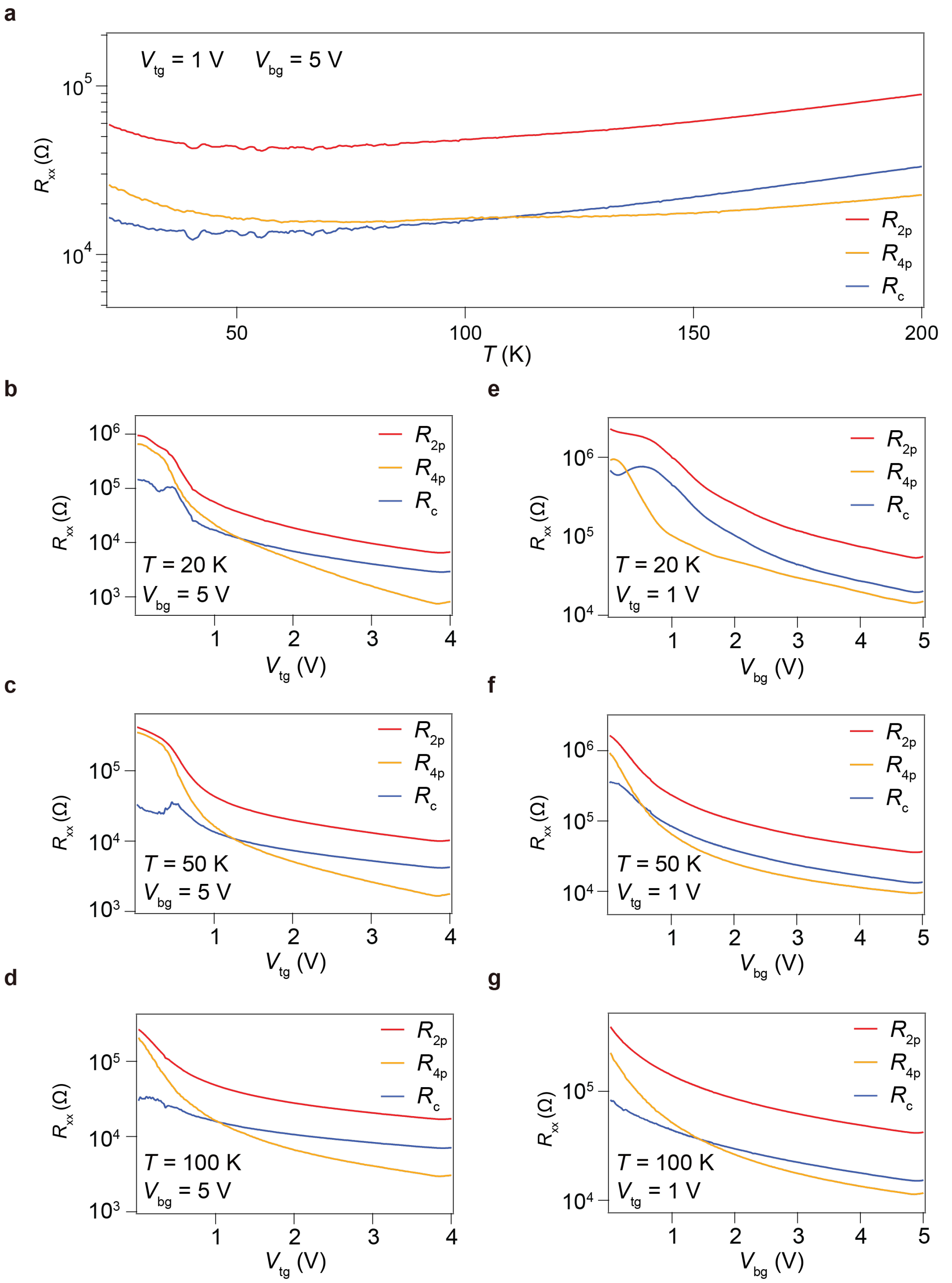

Supplementary Figure 9. Contact resistance of MoS_2 at different temperatures and gate voltages. (a) Two-terminal resistance R_{2p}, four-terminal resistance R_{4p} and the extracted contact resistance R_c for MoS_2 layer in sample-S21 as a function of temperature. The contact resistance R_c for MoS_2 layer in sample-S21 as a function of V_{tg} at (b) $T = 20$ K, (c) $T = 50$ K, and (d) $T = 100$ K, respectively. The contact resistance

R_c for MoS_2 layer in sample-S21 as a function of V_{bg} at (e) $T = 20$ K, (f) $T = 50$ K, and (g) $T = 100$ K, respectively. Note that the contact resistance unit is not normalized by multiplying channel width due to the unconventional drag device geometry. The low contact resistances ($V_{tg} = 1$ V, $V_{bg} = 5$ V) at low temperatures together with linear *I*-*V* curves in Supplementary Figures 6 and 7 suggest that good Ohmic contacts for both MoS_2 and graphene are achieved, enabling the reliable execution of both the drag and transport measurements.

10. Band edge of the window-contacted MoS_2 layer.

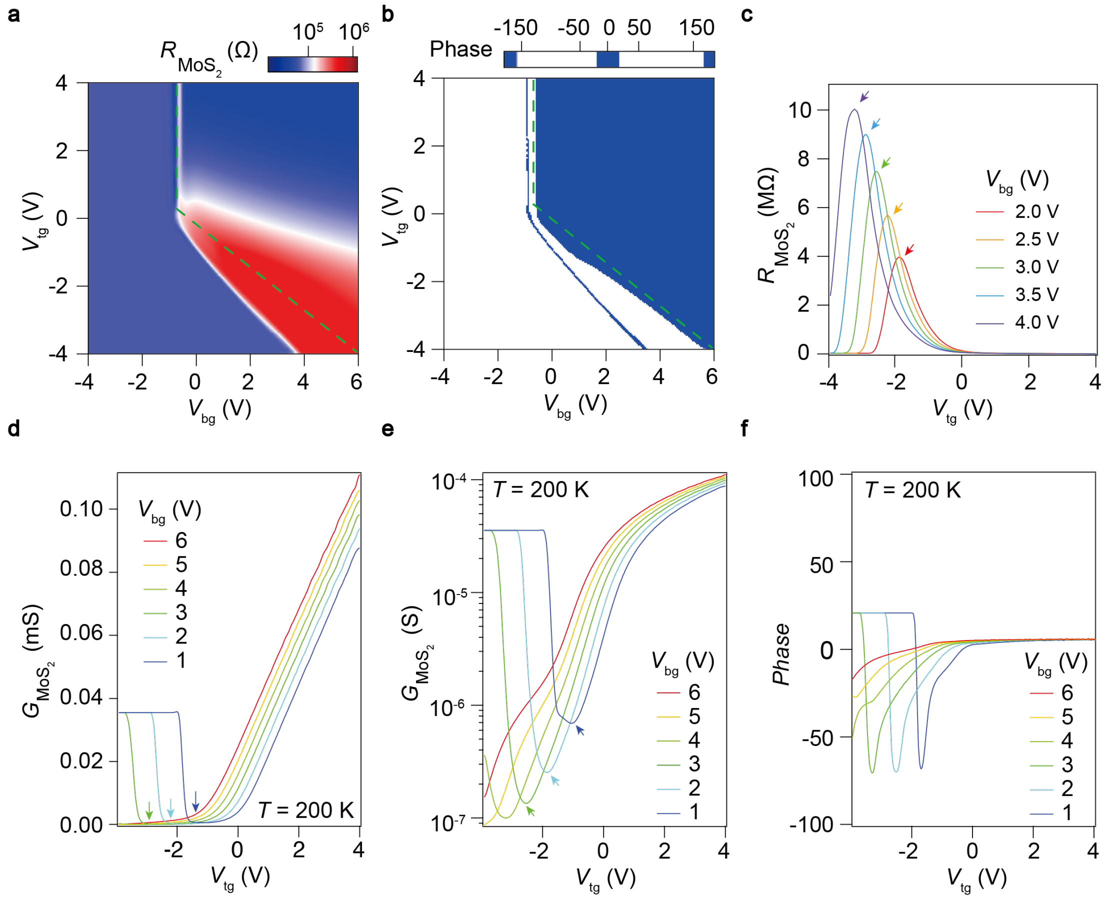

Supplementary Figure 10. The band edge of MoS_2 determined by four-terminal lock-in measurement. 2D maps of the (a) channel resistance and (b) phase of the MoS_2 layer in the parameter space of V_{bg} and V_{tg}. (c) The channel resistance of MoS_2 as a function of V_{tg} at different V_{bg}, with linecuts extracted from the 2D map in (a). The green lines in (a) and (b) indicate the resistance peaks at different gate voltages, which represent the band edge of MoS_2. The green arrows in (c) indicate the different points along the green dashed line in (a). (d) The field effect curves of MoS_2 measured by the ac lock-in method at T = 200 K with different bottom gates. (e) The conductance of MoS_2 as a function of V_{tg} in a semi-logarithmic scale at T = 200 K with different bottom gates. (f) The phase of lock-in amplifier for the corresponding conductance signals in (d) or (e). The colored arrows in these figures indicate the turning points beyond which the intrinsic conductance of MoS_2 can be measured accurately. Due to the semiconducting nature of MoS_2, its resistance increases rapidly as the Fermi level approaches the band gap. This characteristic causes the lock-in amplifier's phase to rise above 10°, and in some cases even change sign, as shown in (f). Therefore, we regard signals acquired under such large phase shifts ($>\pm 10°$) as unreliable and treat them as inaccurate measurements of the intrinsic MoS_2 resistance.

11. Impact of bridge balance on the accuracy of drag signal measurements.

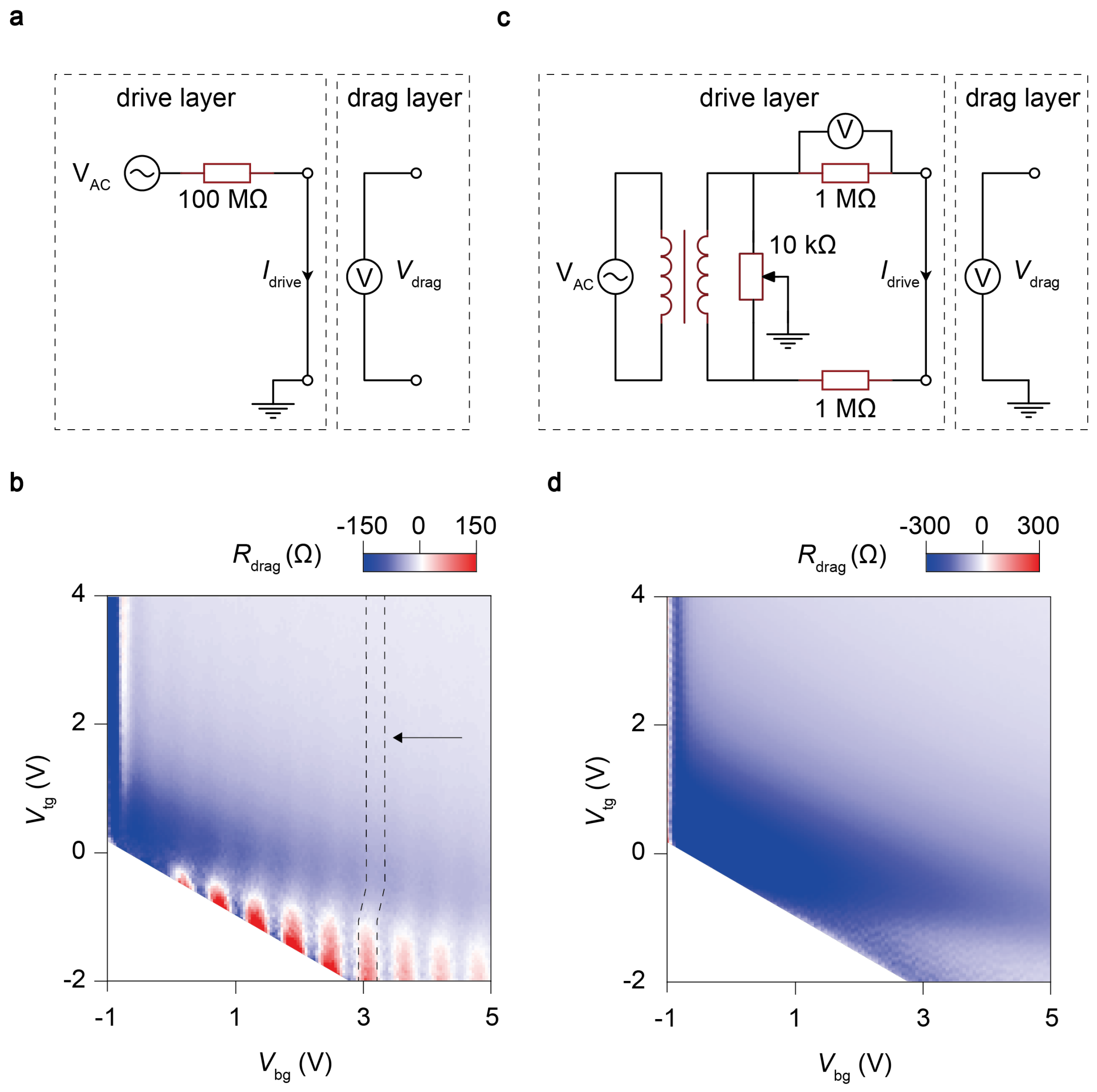

Supplementary Figure 11. Two kinds of setup for measuring Coulomb drag signals. (a) The circuit for a simple lock-in measurement. A current is driven through one layer, while the resulting voltage is measured across the other layer, which remains open-circuited. As a result, the potential at the midpoint of the drive layer reaches $\sim V_{drive}/2$ with respect to ground. Given that the drag layer is grounded, an AC interlayer bias of $\sim V_{drive}/2$ is generated accordingly and produce spurious drag signal, as shown in (b). (c) The circuit for the Coulomb drag measurement. The AC voltage is fed into the bridge circuit through a 1:1 ground-isolating transformer to minimize ground loop. By carefully tuning the variable resistor in the bridge, the AC potential at the center of the drive layer is adjusted to approximately zero, preventing interlayer capacitance coupling and ensuring accurate measurement of the drag signal. The corresponding drag resistivity as a function of V_{bg} and V_{tg} is shown in (d).

12. Identification of the effective Coulomb drag region.

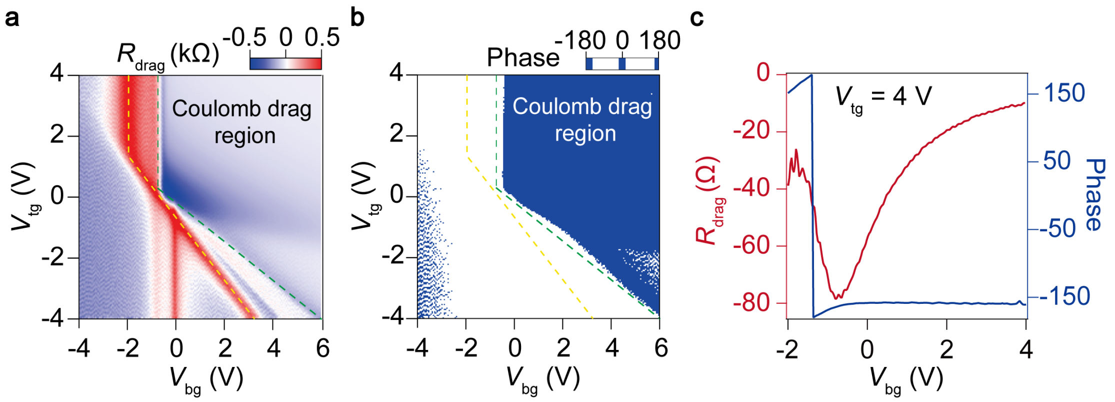

Supplementary Figure 12. Comparison of the measured drag resistance and the corresponding phase for the typical device. (a) The dual-gate map of drag resistance at T = 200 K. (b) The corresponding phase signal of the drag resistance by the lock-in measurement. The yellow and green lines represent the Dirac peak of graphene and band edge of MoS_2, respectively. We can clearly identify the effective Coulomb drag region in the upper-right corner of the 2D map. (c) The drag resistance (red line) and the corresponding phase (blue line) at V_{tg} = 4 V. The result shows the validity of drag signal.

13. The location where the maximum value of R_{drag} occurs.

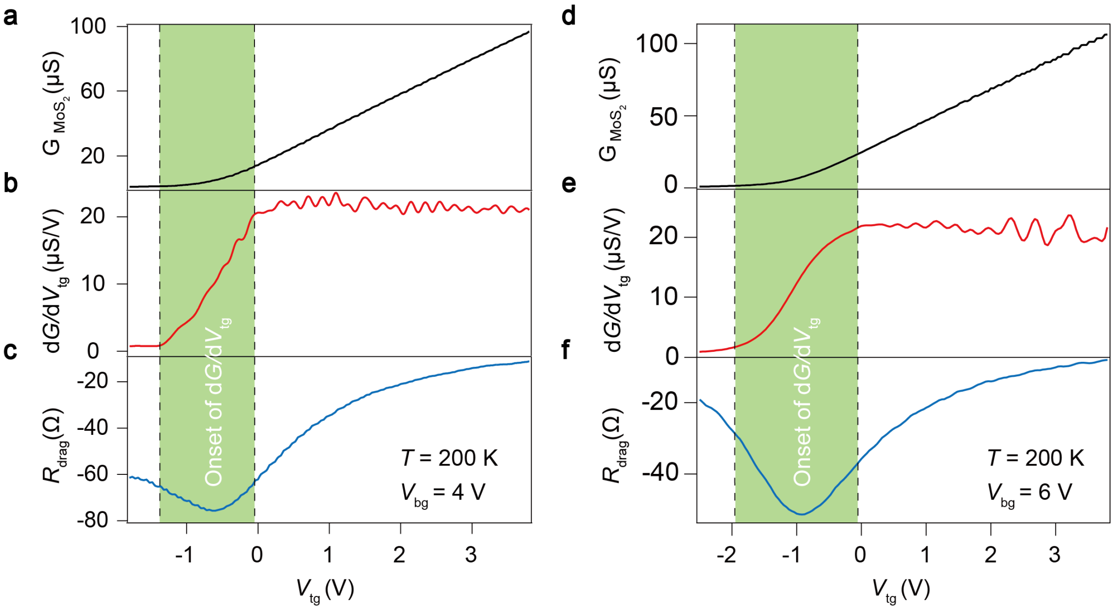

Supplementary Figure 13. The comparison of the V_{tg} dependence of G_{MoS_2}, dG/dV_g and R_{drag}. (a) The conductance of MoS_2 (G_{MoS_2}), (b) the MoS_2 channel conductance derivative with respect to gate voltage (dG/dV_g), and (c) the drag resistance (R_{drag}) of the device as a function of V_{tg} at T = 200 K and V_{bg} = 4 V. (d) G_{MoS_2}, (e) dG/dV_g, and (f) R_{drag} as a function of V_{tg} at T = 200 K and V_{bg} = 6 V. The green regions in the figures indicate the onset of dG/dV_g, within which the maximum value of R_{drag} is found.

14. The leakage current I_g between the drive and drag layers.

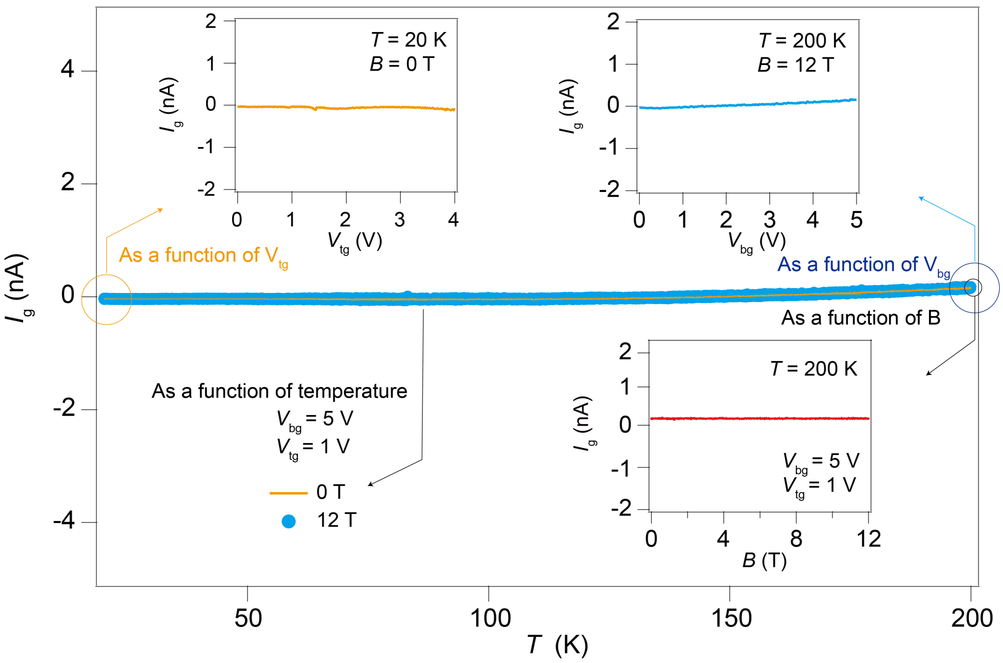

Supplementary Figure 14. The leakage current I_g between the active and passive layers as a function of temperatures at V_{tg} = 1 V and V_{bg} = 5 V when the magnetic field is held for 0 T (yellow line) and 12 T (blue dots), respectively. The insets show I_g as a function of V_{tg}, V_{tg} and magnetic field. The leakage current is negligible during our drag measurements.

15. Linear response of drag voltage to drive current at low temperature.

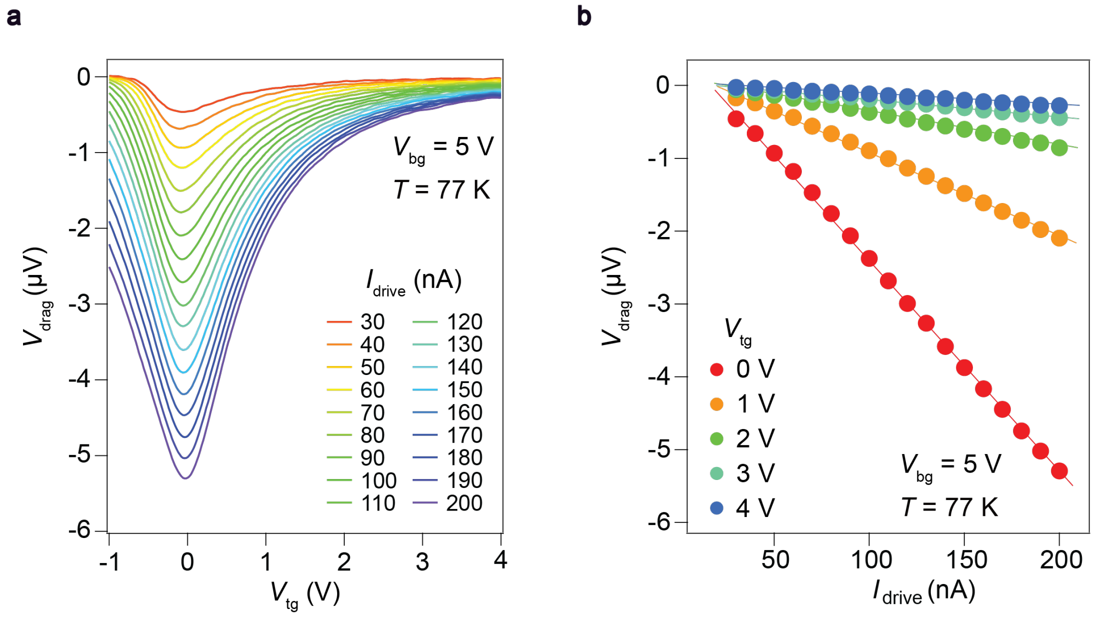

Supplementary Figure 15. (a) The drag response as a function of V_{tg} for various applied drive current at V_{bg} = 5 V and T = 77 K. (b) The relationship between drag response and drive current for different V_{tg}. The colored circles represent the drag voltage data extracted from (a), and the corresponding colored lines show the linear fittings for each represent V_{tg}. The linear relationship between V_{drag} and I_{drive} still holds at low temperature for different V_{tg}.

16. Onsager reciprocity in the drag system.

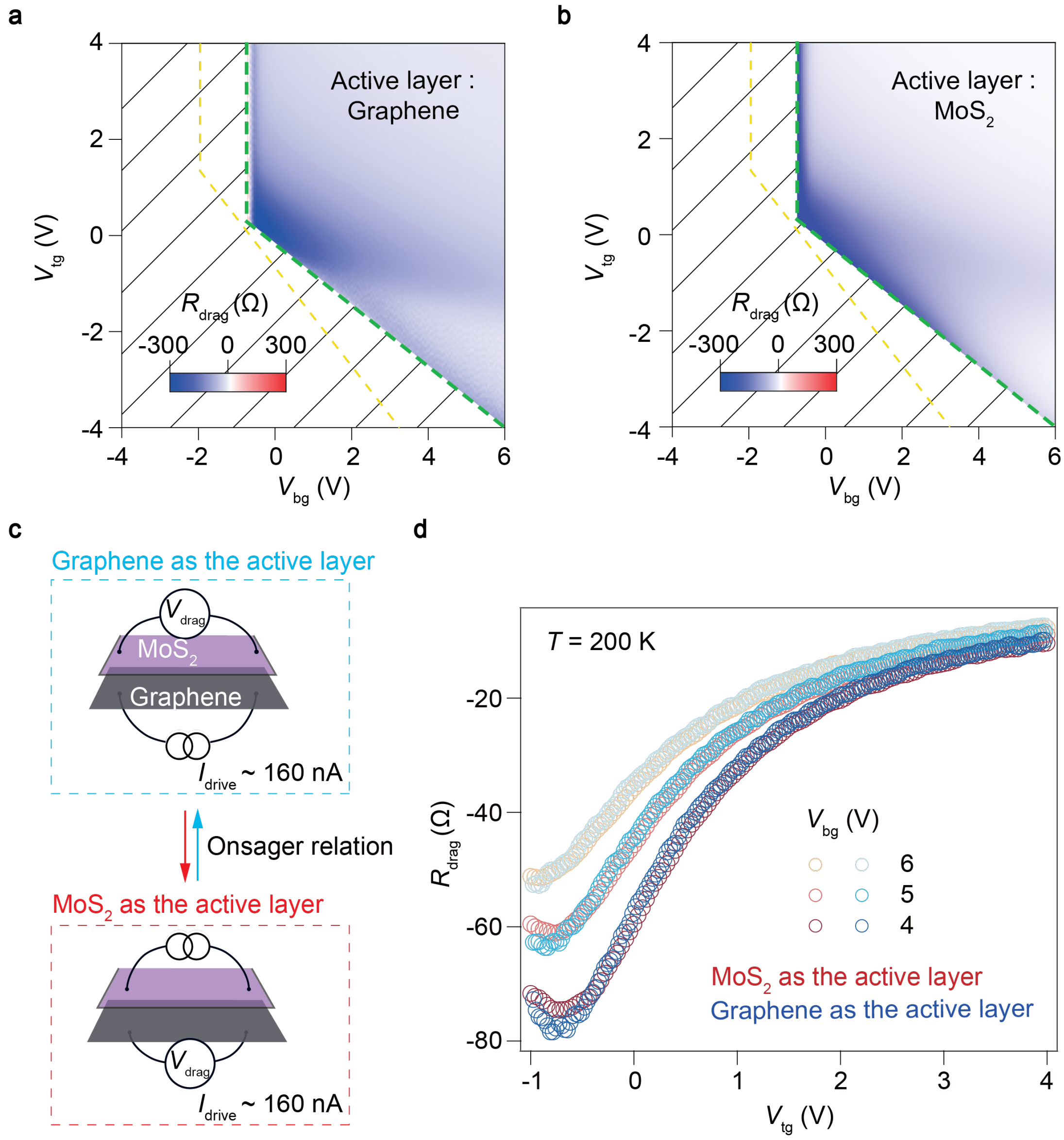

Supplementary Figure 16. Dual-gate mapping of the drag resistance when (a) the graphene layer and (b) the MoS_2 layer serves as the active layer. Certain regions in the maps (a) and (b) are masked because they fell out of phase with the lock-in amplifier, as seen in Supplementary Figure 12. (c) The schematic of Onsager relation with the active (passive) layer alternated in the drag system. (d) The drag resistance as a function of V_{tg} at different V_{bg}, measured at $T = 200$ K. The red and blue circles represent serving MoS_2 and graphene as the active layer, respectively.

17. Determination of carrier density by classic Hall effect.

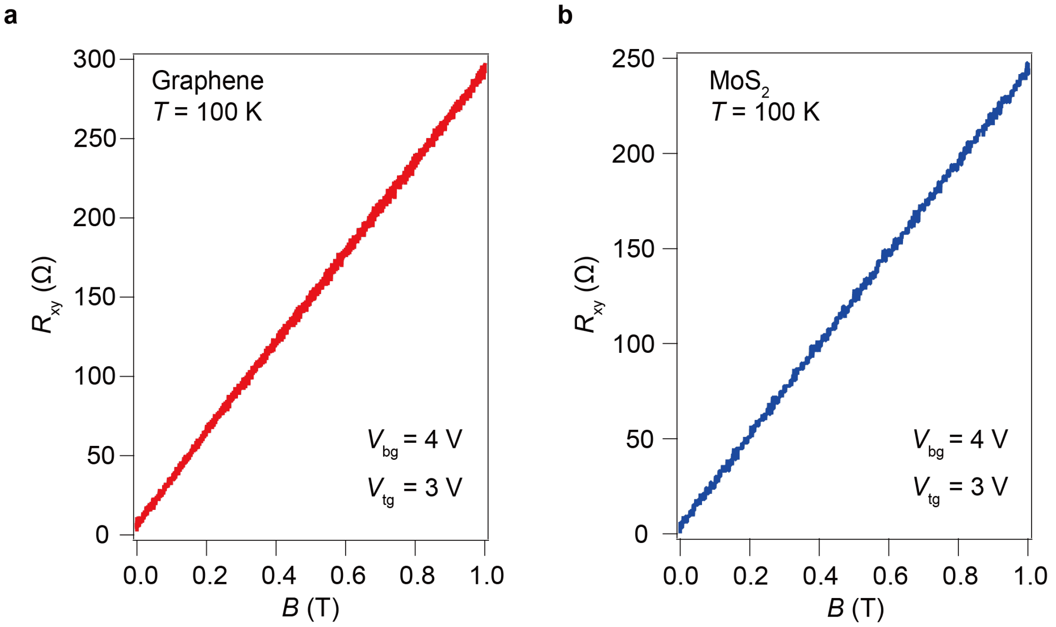

Supplementary Figure 17. The measured R_{xy} as a function of magnetic field for graphene and MoS_2 at 100 K. The transverse resistance of (a) graphene and (b) MoS_2 as a function of B at $V_{bg} = 4$ V and $V_{tg} = 3$ V. We can calculate the carrier densities of both the graphene and MoS_2 layer by extracting Hall coefficient from the linear R_{xy}-B curves.

18. Hall carrier density and Hall mobility of each layer for different temperatures and gate voltages.

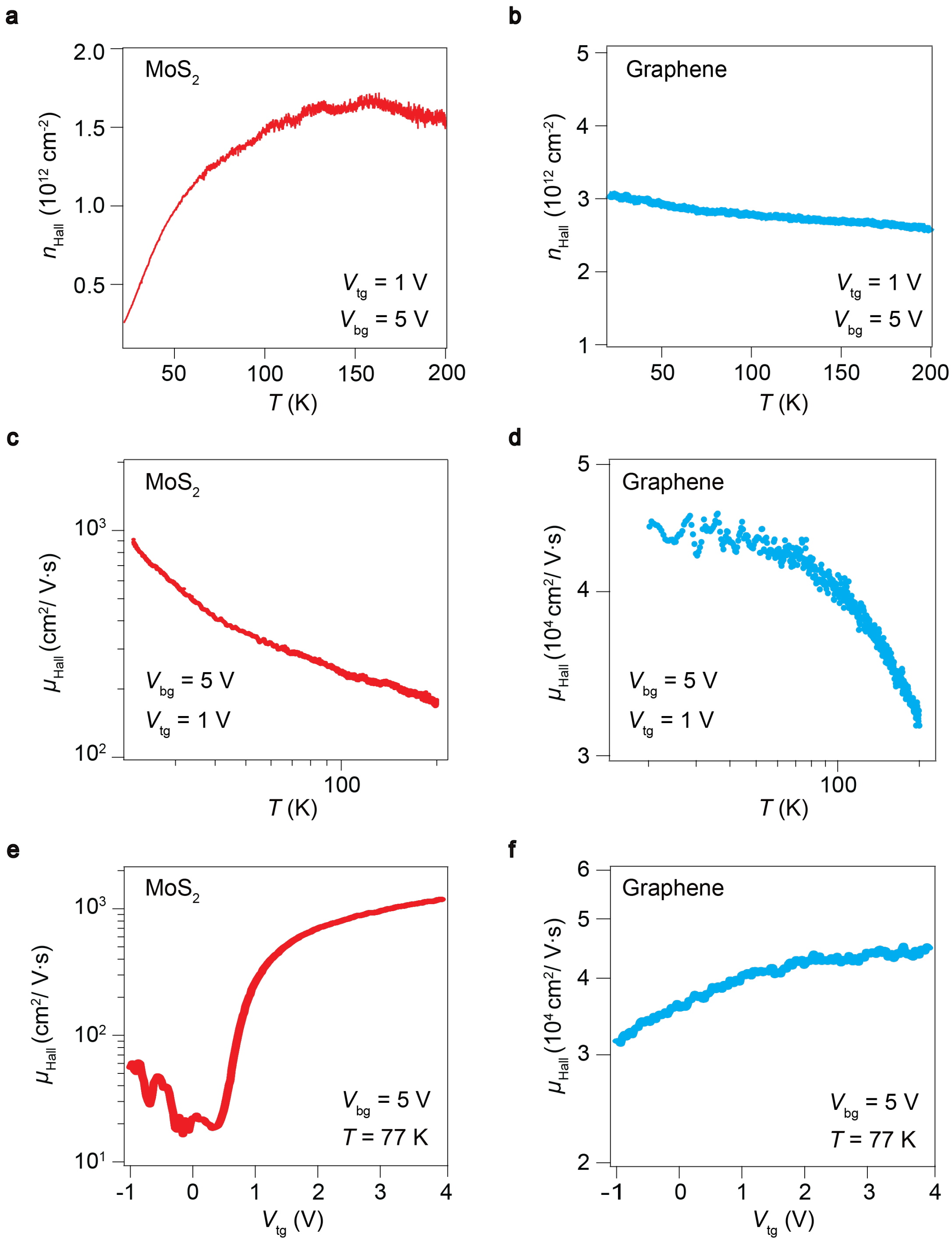

Supplementary Figure 18. The Hall carrier density as a function of temperature in (a) MoS_2 and (b) graphene at V_{tg} = 1 V and V_{bg} = 5 V, respectively. The Hall mobility in (c) MoS_2 and (d) graphene at V_{tg} = 1 V and V_{bg} = 5 V for different temperatures, respectively. The Hall mobility as a function of V_{tg} in (e) MoS_2 and (f) graphene at V_{bg} = 5 V and T = 77 K.

19. Field-effect mobility of each layer for different temperatures and gate voltages.

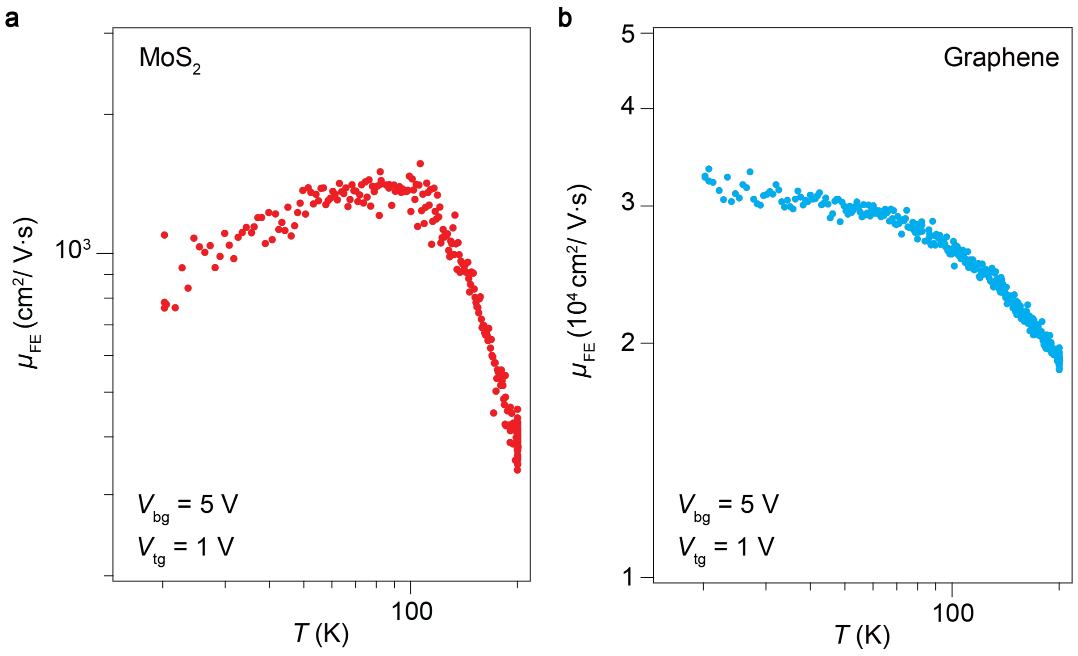

Supplementary Figure 19. The field-effect mobility of (a) MoS_2 and (b) graphene for various temperatures at $V_{tg} = 1$ V and $V_{bg} = 5$ V, respectively.

20. Onsager reciprocity relation of magneto-drag resistance.

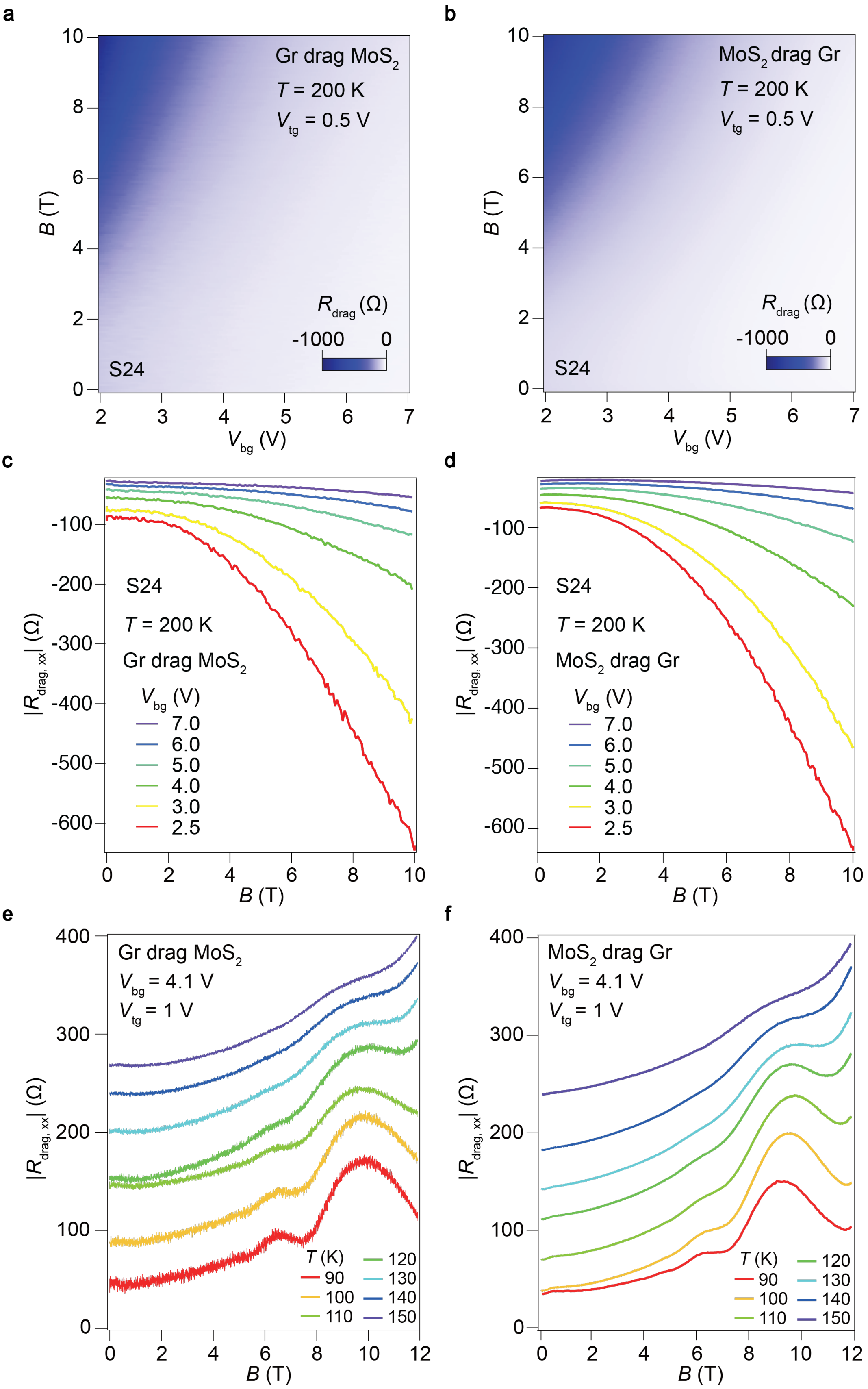

Supplementary Figure 20. Dual-gate maps of the drag resistance of sample-S24 at T = 200 K with (a) the graphene layer and (b) the MoS_2 layer serving as the drive layer. The drag resistance versus magnetic field for varying V_{bg} when (c) graphene and (d) MoS_2 serving as the drive layer, respectively. The drag resistance as a function of magnetic field at the same gate voltage (V_{bg} = 4.1 V and V_{tg} = 1 V) but different low temperatures, for reciprocal layer configurations: (e) graphene drag MoS_2 and (f) MoS_2 drag graphene. The curves in (e) and (f) are vertically shifted for better visualization. Note that the curves in (e) and (f) for reciprocal configurations do not overlap perfectly; this deviation may be induced by disorder within the device.

21. Magneto-drag behavior in semiconductor-semimetal bilayers at 5 T and 30 K.

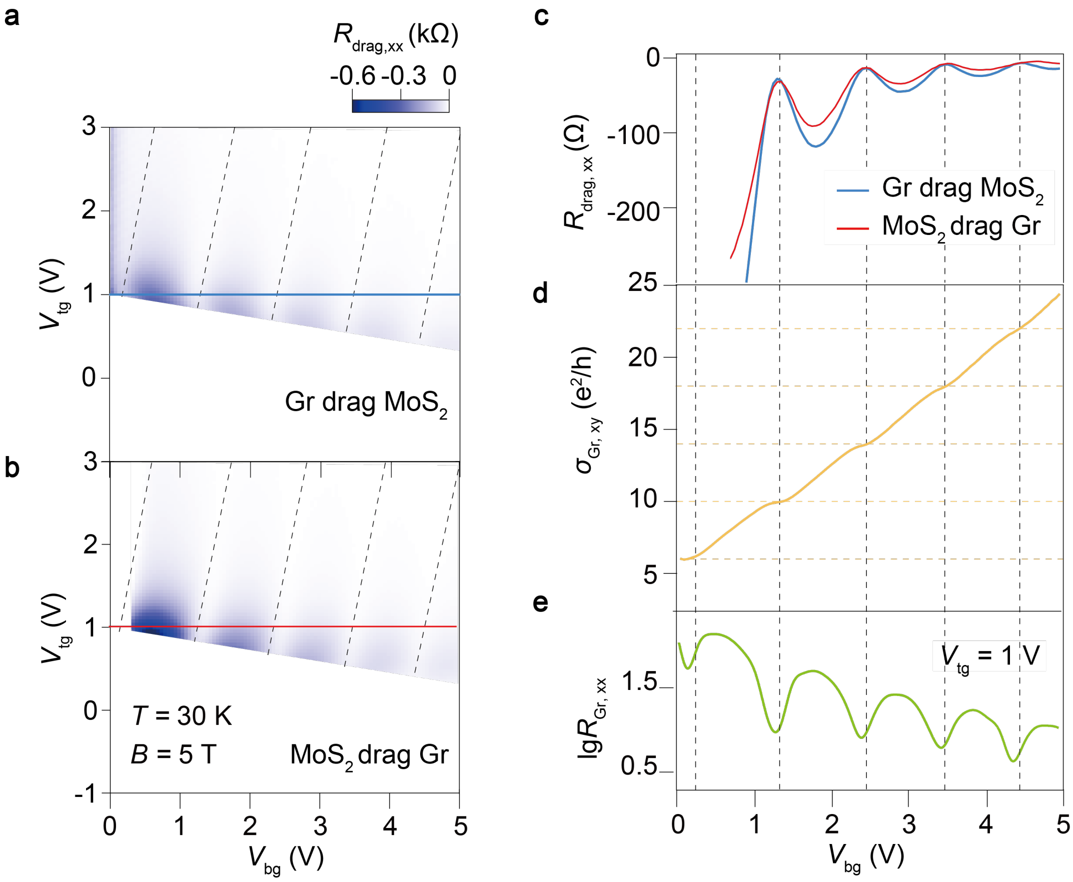

Supplementary Figure 21. Magneto-drag responses in semiconductor-semimetal double layers at 5 T and 30 K. The dual-gate maps of magneto-drag resistance in the system, with (a) MoS_2 and (b) graphene as the active layer, respectively. At lower temperature (T= 30 K), Landau levels develop in the graphene layer, and the Onsager relation in the drag system is fulfilled only when MoS_2 enters the metallic state. (c) Line profiles of $R_{drag, xx}$ with graphene (blue) and MoS_2 (red) as the active layer, respectively. (d) Transverse conductivity and (e) longitudinal resistance of the graphene channel over the same gate range as in (c).

22. Drag oscillations versus 1/*B* show the same spacing as graphene's SdH oscillations.

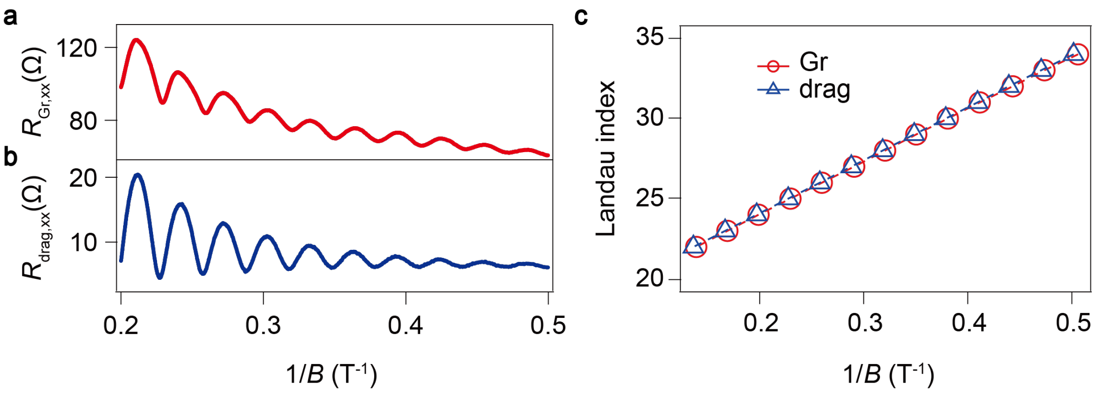

Supplementary Figure 22. Drag oscillations exhibit the same 1/*B* periodicity as SdH oscillations in graphene. Longitudinal resistance of (a) graphene channel and (b) drag versus 1/*B* at 20 K with V_{bg} = 5 V and V_{tg} = 1 V. (c) The minima of $R_{drag,\ xx}$ are consistent with the Landau index of graphene, as obtained from (a) and (b).

23. The visible oscillations of the magneto-drag signal at high temperatures in sample S24.

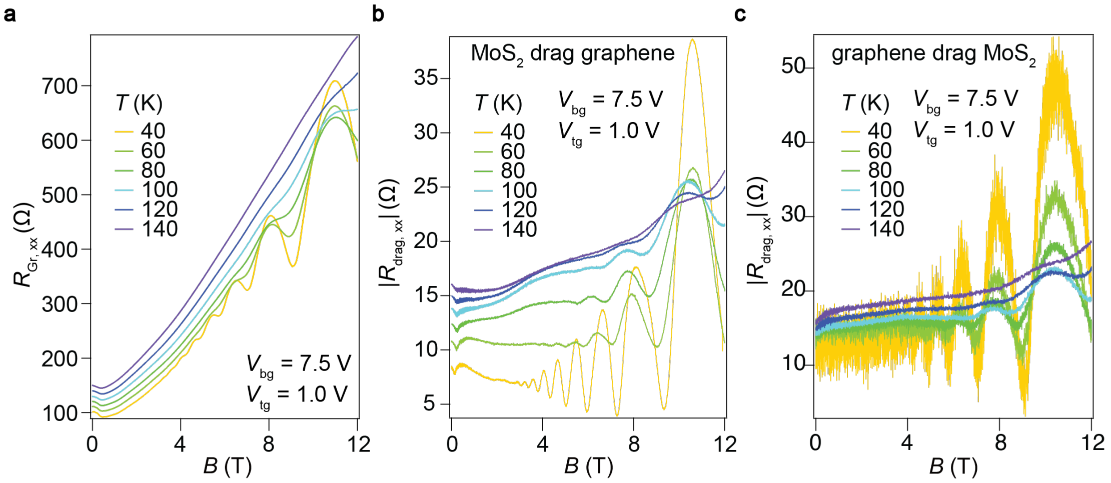

Supplementary Figure 23. The comparison of intrinsic SdH oscillations of the graphene layer and magneto-drag resistance at different temperatures in sample S24. (a) R_{xx} of graphene as a function of magnetic field for different temperatures. $R_{drag,\ xx}$ as a function of magnetic field for different temperatures, where (b) MoS_2 and (c) graphene serve as the drive layer. All data are taken at V_{bg} = 7.5 V and V_{tg} = 1 V. The amplitudes of oscillations of drag resistance are much larger than those of graphene layer at high temperatures.

24. The drag response for samples with different spacer thickness *d*.

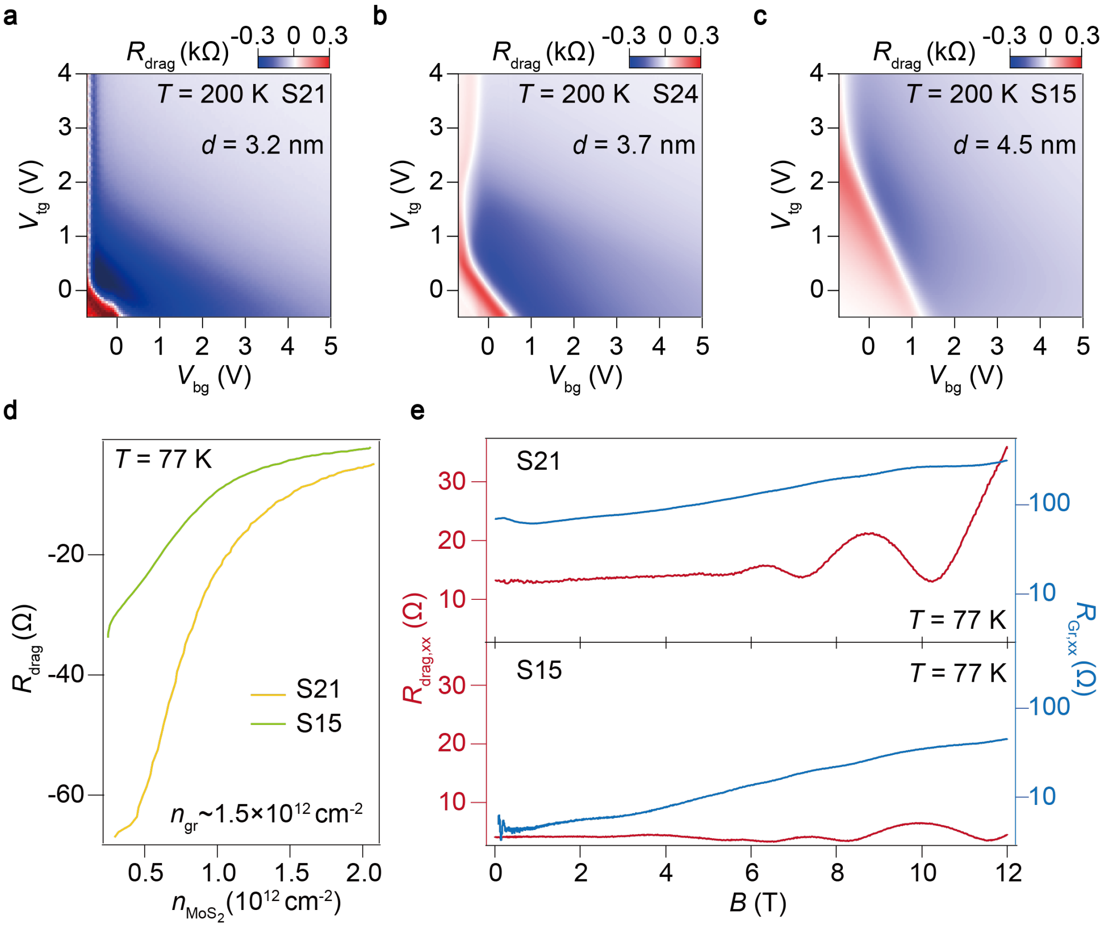

Supplementary Figure 24. The drag resistance as a function of V_{tg} and V_{bg} for three samples, labeled (a) S21, (b) S24 and (c) S15, which feature different interlayer h-BN spacer thicknesses. (d) The drag resistance as a function of carrier density in MoS_2 for samples S21 and S15 with a same carrier density in graphene at 77 K. (e) A comparison of intrinsic resistance of graphene and drag resistance as a function of magnetic field between sample S21 and S15. The data is measured at 77 K with the same fixed carrier density in both the graphene and MoS_2 layer.
